# Surgical outcomes of the removal of posterior segment metallic intraocular foreign bodies

**DOI:** 10.1186/s12886-020-01535-5

**Published:** 2020-07-06

**Authors:** Kai-Ling Peng, Ya-Hsin Kung, Pyn-Sing Hsu, Tsung-Tien Wu

**Affiliations:** 1 Department of Ophthalmology, Kaohsiung Veterans General Hospital, 386, Ta-Chung 1st Road, Kaohsiung, 813 Taiwan, Republic of China; 2 School of Medicine, National Yang-Ming University, Taipei, Taiwan, Republic of China

**Keywords:** Posterior segment, Metallic intraocular foreign bodies, Vitrectomy, Scleral buckling, Tamponade, Retinal detachment, Endophthalmitis

## Abstract

**Background:**

Posterior segment metallic intraocular foreign bodies (IOFBs) are a leading cause of visual morbidity and blindness, especially among young and middle-aged working populations. Here, we aimed to evaluate the surgical outcomes of the removal of such IOFBs that result from injuries.

**Methods:**

In this retrospective study, 39 patients injured by metallic posterior segment IOFBs and who underwent primary repair procedures, vitrectomies, and IOFBs removal with or without procedures for traumatic cataract removal, scleral buckling and intraoperative tamponade application from January, 2008 to January, 2019. We analyzed the preoperative, intraoperative and postoperative related factors that affect the final visual outcomes.

**Results:**

The mean age of the 39 patients was 40.51 ± 12.48 years with the male being predominent (100%).The mean preoperative vision measured 1.50 [Snellen Equivalent (SE), 20/645] ± 1.12 logMAR with the mean final vision measuring 0.93 (SE, 20/172) ± 1.09 logMAR. The related factors that were determined to affect the final visual outcomes included preoperative vision (*P* = 0.025), IOFB-related macula injuries (*P* = 0.001) and the development of postoperative complications (*P* = 0.005) especially retinal detachment (*P* = 0.002) with the mean final vision measuring 2.12 (SE, counting finger to hand motion) ±1.23 logMAR. Concerning the preoperative signs, the patients with preoperative endophthalmitis also obtained poor mean final vision measuring 1.30 (SE,20/400) ± 1.40 logMAR.

**Conclusion:**

IOFB-related macula injuries and postoperative retinal detachment were important related factors of poor final visual prognoses in cases involving posterior segment metallic IOFBs. Removing IOFB as early as possible may prevent preoperative endophthalmitis which could lead poor final visions even without significance.

## Background

Posterior segment metallic intraocular foreign bodies (IOFBs) are one of leading causes of visual morbidity and blindness, especially among the young and middle-aged working populations [1, 2]. They present very distinct surgical challenges since these objects travel at high speeds serially through the ocular organs, moving from the cornea into the lens and, vitreous cavity and finally impacting the retina. For the removal of IOFBs, all the tissues along the route through which they passed should be repaired in an order starting from the external organs first and then moving onto internal organs consecutively. Therefore, an extensive amount of time is consumed simultaneously performing several surgeries. The potential serious consequences that are associated with posterior segment metallic IOFBs include traumatic cataract with lens rupture, vitreous hemorrhage (VH), retinal detachment (RD) and endophthalmitis. Based on the surgical conditions and treatment plan devised by the surgeons, it is decided whether all these complications should be resolved in one surgery involving the removal of the IOFBs or whether the procedure should be divided into two or three surgeries to complete the treatment. However, RD associated with proliferative vitreoretinopathy (PVR), endophthalmitis, and secondary choroidal neovascular (CNV) membrane and sympathetic ophthalmia (SO) are also possible complications of posterior segment metallic IOFBs. Therefore, visual prognoses related to posterior segment metallic IOFBs might obviously vary depending on several factors that, involve the position of the metallic IOFB-related injury at the corneal entrance site and on the retinal bump, complexity of any associated issues and severity of resulting complications. Therefore, in this study, we aimed to evaluate the surgical outcomes of the removal of metallic posterior segment IOFBs following injuries.

## Methods

In this retrospective study, we followed the tenets of the Declaration of Helsinki, and the study was approved by the hospital’s Institutional Review Board. We reviewed the medical records of consecutive patients who sustained injuries to the posterior ocular segment involving metallic IOFBs between January 2006 and December 2018. The inclusion criteria were as follow: injured patients with posterior segment metallic IOFBs who underwent vitrectomies and procedures involving the removal of metallic IOFBs. The exclusion criteria were patients with anterior segment IOFBs, those who underwent enucleation as the primary treatment modality, those with posterior segment non-metallic IOFBs, such as glass and wood and those who were followed up for less than 1 month.

All the injured patients were subjected to an enquiry that recorded a detailed account of their trauma history and to a complete ophthalmological examination at the presentation that included measurements of Snellen best-corrected visual acuity (BCVA), slit-lamp biomicroscopy, external eye and color fundus photography, and indirect ophthalmoscopy. BCVA was measured using Snellen charts, and subsequently converted into logarithm of the minimal angle of resolution (logMAR) values for these statistical analysis [3]. Before operating the patients, the diagnosis of posterior segment IOFBs was confirmed by computed tomography (CT) imaging scans.

We recorded each patient’s baseline demographic and medical information, including age, sex, preoperative BCVA, final BCVA and follow-up duration. Data regarding the IOFBs including the sizes of the metallic IOFBs, wound sizes at the entry points of the IOFBs, and interval between the sustenance of the IOFB-related injuries until the removal of the IOFBs were also recorded. In addition, we also collected information regarding the preoperative ocular features involving the anterior segments such as whether or not the following were present: corneal lacerations involving the cornea or sclera, iris injuries and lens opacities with ruptures; the information regarding the posterior segments included whether there were preoperative signs and the location of the exact site of the IOFB impact on the retina, inside the macula or on the peripheral retina. The intraoperative factors that we assessed consisted of whether either concomitant sclera buckling (SB) or the application of a tamponade were required when the surgery was completed base on the presence or absence of concomitant retinal breaks or RD following metallic IOFB-related injuries. Furthermore, we recorded postoperative complications involving the posterior segments following the surgical procedures.

We further subdivided the preoperative signs to include preoperative VH, endophthalmitis and RD; the intraoperative factors were subdivided into where the application of a tamponade with or without gas (sulfur hexafluoride gas or perfluoropropane) and silicon oil and concomitant SB were required; and the postoperative complications associated with the posterior segments following the surgical procedures were subdivided into signs such as postoperative RD, macular CNV, macular pucker, macular atrophy and scarring. The criteria of preoperative endophthalmitis were hypopyon and vitreous opacity.

The surgeries of all the patients were performed under general anesthesia by well-trained retinal specialists. All the surgical procedures included the following steps: cornea laceration wound suturing, three-port 20 or 23 gauge pars plana vitrectomy (PPV), a core vitrectomy, and the removal of all vitreous traction around the IOFBs. In cases in which the surgical view was significantly blurred due to the opacity resulting from traumatic cataract, a simultaneous cataract surgery was performed to facilitate the clear visualization of the posterior segments. The IOFBs were mobilized and removed using intraocular forceps or intraocular magnets after enlargement of the sclerotomy wound. Following IOFBs removal, a peripheral retinal examination was carefully performed using sclera depression to detect if there were any retinal breaks. The retinal breaks that were related to the IOFB injury were treated with endolaser therapy, photocoagulation, or cryotherapy. In patients with retinal breaks or RD resulting from IOFB-related injuries, an injection of long-lasting gas or silicon oil with concomitant SB was administered at the end of the surgery. Simultaneously, an intravitreal injection of antibiotics containing ceftazidime 2.25 mg/0.1 ml and vancomycin 1 mg/0.1 ml was administered.

To identify the potential factors associated with the final visual outcomes in patients with posterior segment metallic IOFBs, the clinical data were statistically analyzed. The visual acuity (VA) was measured using a standard Snellen acuity chart and was converted to a (logMAR) units for the statistical analysis.^1^ We used linear regression to compare the final visual outcomes (final BCVA logMAR) with the continuous variables including age, IOFB sizes, wound sizes, and follow-up time. For categorical variables with two factors, we used an independent *t* test to compare the differences in the final visual outcomes in relation to the time interval between the onset of the IOFB injury until IOFB removal, wound position in the cornea or sclera, and the presence or absence of the following: iris injuries, lens trauma, retinal injuries in the macular or peripheral regions, preoperative signs, the need for concomitant SB and/or an intraoperative tamponade and postoperative complications. Data were analyzed using the IBM SPSS statistical software version 20.0 (Armonk, NY). A *P* level of < 0.05 was considered significant.

## Results

During the study period, a total of 39 eyes met the inclusion criteria and underwent surgery for the removal of metallic IOFBs. Men accounted for 100% (39/39) of the patients in this study. Among them, 99.97% (38/39) were injured while working, and 2.56% (1/39) were inured by firecrackers. The most common task that resulted in inuries was mechanical metal grinding involving 17.95% (7/39) of the patients. Other tasks included hammering on metal (15.38%) (6/39), weeding (7.69%) (3/39), and drilling (5.13%) (2/39). The mean age was 40.51 ± 12.48 years (range: 13 to 63). The mean preoperative VA was 1.50 [Snellen Equivalent (SE), 20/645] ± 1.12 logMAR (range: 0 to 3). The mean final VA was 0.93 (SE, 20/172) ± 1.09 logMAR (range: 0.17 to 3). The mean follow-up time was 21.18 ± 21.29 months (range: 1 to 108 months). The mean IOFB size was 12.24 ± 22.22 mm^3^ (range: 0.3 to 126). The mean wound size was 2.26 ± 1.39 mm (range: 0.1 to 6.0). Table 1 summarizes the baseline data of the patients injured by metallic IOFBs. Among the 39 patients, 25 (64.10%) had an injured right eye, 19 (48.72%) underwent surgeries with an interval of more than 24 h between sustaining the IOFB injury and the surgyery, 7 (17.95%) had an IOFB entrance point on the sclera, 26 (66.67%) had iris injuries, 26 (66.67%) had simultaneous lens trauma, 11 (28.21%) experienced IOFBs impact on the macula, 21 (53.85%) underwent either the application of an intraopearative tamponade at the end of surgery or concomitant SB and 13 (33.33%) developed postopeartive complications.

**Table 1:** The baseline data of patients injured by metallic IOFBs

	Mean ± SD N. (%)	Final BCVA (logMAR) Mean	*P*
Age (years)	40.51 ± 12.48		0.460^b^
Preoperative BCVA (logMAR)	1.50 ± 1.12	0.350^+^	0.025^* b^
Final BCVA (logMAR)	0.93 ± 1.09		
Follow-up (Months)	21.18 ± 21.29		0.072 ^b^
IOFB size (mm^3^)	12.24 ± 22.22		0.948^b^
Wound size (mm)	2.26 ± 1.39		0.107 ^b^
Eye (OD/OS)	25 (64.10)	1.05/0.72	0.515^a^
Interval (> 24 h/< 24 h)	19 (48.72)	1.05/0.83	0.295^a^
Wound position (sclera/cornea)	7 (17.95)	0.96/0.84	0.929^a^
Iris injury (yes/no)	26 (66.67)	0.94/0.93	1.000^a^
Lens trauma (yes/no)	26 (66.67)	0.99/0.83	0. 895^a^
Retina injury (macula/periphery)	11 (28.21)	1.79/0.60	0.001^* a^
Preoperative signs (yes/no)	29 (74.36)	1.06/0.57	0.174^a^
Scleral buckle or tamponade (yes/no)	21 (53.85)	0.75/1.15	0.865^a^
Postoperative complications (yes/no)	13 (33.33)	1.70/0.55	0.005^* a^

^*^P < 0.05, ^a^ Mann- Whitney U test, ^b^ linear regression; ^+^correlation coefficient; *N*., number; *%* percentage; *SD* standard deviation; *BCVA* best-corrected visual acuity; logMAR, logarithm of minimum angle of resolution

### Facotrs affecting visual prognoses

The related factors that potentially influenced the final visual outcomes consisted of the preoperative VA (*P* = 0.025), retinal area injured by IOFBs (*P* = 0.001) and presence of postoperative complications (*P* = 0.005). Macular injury worsened the mean final VA 1.79 (SE, 20/1237) ± 1.08 logMAR and peripheral retina injury got better mean final VA 0.6 (SE, 20/80) ± 0.92 logMAR. Presence of postoperative complications worsened the mean final VA 1.70 (SE, 20/1003) ± 1.22 logMAR while without postoperative comlications got better mean final VA 0.55 (SE, 20/71) ± 0.81 logMAR. The correlation between the final visual outcomes and preoperative VA was significant (*P* = 0.025).

### Subdivided factors affecting visual prognoses

Based on the preoperative signs subdivisions, the use of an intraoperative tamponade and concomitant SB, and posteropative complications, we further analyzed the final visual outcomes and summerized them in Table 2. Regarding the preoperative signs, the most commonly observed signs was preoperative VH with 66.67% (26/39) of the patients. Concerning intraoperative tamponade and concomitant SB requirements, there were 21 (53.85%) patients required an intraoperative tamponade at the end of the surgery and 7 (17.95%) patients undwent concomitant SB. The most common postoperative complication was RD affecting 20.52% (8/39) of the patients. There was one patient who developed macular CNV and another one who developed macular pucker following the surgeries for IOFB removal. Both of these patients were later treated with the final results indicating no further comlications. Lastly, 7.69% (3/39) of the patients developed postoperative complications including macular atrophy and macular scarring.

**Table 2:** The relationships between final visual outcomes with subdivided related factors of preoperative signs, scleral buckle or temponade and postoperative complications

	N. (%).	Final BCVA (logMAR) Mean	*P*
Preoperative signs			
Vitreous hemorrhage (yes/no)	26 (66.67)	1.10/ 0.61	0.178^a^
Endophthalmitis (yes/no)	6 (15.38)	1.30/ 0.87	0.690 ^a^
Retinal detachment (yes/no)	2 (5.13)	1.15/ 0.93	0.356 ^a^
Scleral buckle or temponade			
Intraoperative tamponade (yes/no)	21 (53.85)	0.75/ 1.15	0.865 ^a^
Concomitant scleral buckle (yes/no)	7 (17.95)	1.03/ 0.92	0.419 ^a^
Postoperative complications			
Retinal detachment (yes/no)	8 (20.52)	2.12/ 0.63	0.002^*a^
Macular CNV and pucker (yes/no)	2 (5.13)	0.05/0.99	0.097 ^a^
Macular atrophy and scar (yes/no)	3 (7.69)	1.67/0.88	0.089 ^a^

^*^P < 0.05, ^a^ Mann-Whitney U test, *N*, number; *%* percentage; *BCVA* best-corrected visual acuity; *logMAR* logarithm of minimum angle of resolution

Although the preoperative sign divisions were not significantly related to the final visual outcomes, preoperative endophthalmitis resulted in a worse final mean VA of 1.30 (SE, 20/400) ± 1.41 logMAR, and preoperative RD resulted in a poor final mean VA of 1.15 (SE, 20/283) ± 0.21 logMAR. Contrarily, performing intraoperative SB or applying a tamponade at the end of surgery were also not significant to the final visual outcomes but patients who required intraoperative tamponade obtained a better final mean VA of 0.75(SE, 20/115) ± 0.88 logMAR. Concerning the postoperative complication subdivisions, postoperative RD (*P* = 0.002) was the most important related factor associated with the final visual outcomes and produced a poor mean final VA of 2.12 (SE, counting finger to hand motion) ± 1.23 logMAR. Moreover, postoperative macular atrophy and scarring resulted in a poor mean final VA of 1.67 (SE, 20/925) ± 0.35 logMAR.

## Discussion

Several previous reports have discussed metallic posterior segment IOFBs that may result from occupational or work-related accidents especially in the case of individual working without protection goggles. Because multiple ocular tissues are involved in this injuries, it is necessary to perform primary repair procedures to treat all the involved ocular tissues, to remove posterior segment metallic IOFBs, and to manage the associated conditions simultaneously. Concerning the final visual outcomes, it is very complicated to analyze the risk factors that may result in bad visual prognoses.

In the current study, the predictive factors associated with the final visual outcomes for posterior segment IOFBs were subsequently analyzed using different statistical approaches [4–7]. Ehlers et al. reported that the location of the posterior segment IOFBs, a younger age, and increased wound length are significant predictors for poor final visual outcomes [8]. Yang et al. demonstrated that with further adjustments for age and the initial VA, the occurrence of RD and larger IOFB dimensions independently predicted worse final visual outcomes [9]. In our study, the final visual outcomes were related to factors including the preoperative vision, IOFB-related macula injuries, which is the same as the findings of Ehlers et al., and the correlation with the presence of postoperative complications, especially RD, was the same as Yang et al’s findings. We found that IOFB size was not significant to the prognosis of the visual outcome. We speculated that the possible reason for this was that the random facets of the IOFBs that directly bumped into the ocular surface or retina at high speeds resulted in a correlated wound size which may not correspond to the longest dimensions of the IOFB-related injured ocular surface and retina.

Ehlers et al. also reported that 27% of patients with metallic IOFBs required secondary surgery for RD repair [8] whereas Azad et al. revealed that a concomitant SB procedure at the time of the IOFB removal surgery might reduce the risk of RD by 24% [10]. In our study, we further analyzed the intraoperative factors associated with concomitant SB procedures and an additional tamponade applied at the end of the surgical procedures in some cases, at the time of IOFB removal based on the retinal conditions. Ours results revealed a relatively lower incidence of postoperative RD that affected 20.52% (8/39) of the patients. Among these patients, 50% (4/8) required an intraoperative tamponade at the end of surgery, and 37.5% (3/8) also underwent concomitant SB; however, they still developed postoperative RD, which may result unfavorable final visual outcomes. Further studies should be conducted to investigate the intraoperative factors that could prevent secondary RD. On the other hand, it was interesting to uncover that the patients who required an intraopeartive tamponade, which is not significant to the final visual outcomes, obtained better mean final visions results than those who did not require a tamponade. The possible reason for this may be that maintaining the concentrate of blood, bacteria, and free metal particles at a relatively low level following air-fluid exchange at the end of surgery could reduce inflammation and toxicity in the retina.

Moreover, poor VA at presentation has previously been reported to be an important predictive factor for poor visual outcomes [4]. However, some studies have demonstrated that there were no statistically significant correlations between patient groups with poor preoperative VA and those with poor final VA that they proposed many damaged structures including the cornea, lens, or retina cane be treated medically and surgically [11]. In our study, the preoperative VA was one of the significant factors that determined the final visual outcomes. We considered that some of the related factors associated with preoperative vision, such as preoperative VH, which could be treated either medically or surgically resulted in poor preoperative vision; however, these factors may not affect the final visual outcomes. Similarly, other related factors, such as larger IOFB sizes and wound sizes also result in poor preoperative vision because of higher rates of iris injury and lens trauma; however, traumatic hyphema and cataract could be treated and may not affect the final visual prognosis.

It has been proposed that the risk of endophthalmitis decreases with early IOFB removal [12–14]. A previous study has revealed the incidence of endophthalmitis was 9.1% in patients whose mean time of IOFB removal was 30.7 days, but this was observed in patients who were routinely administered intravitreal injection before IOFB removal [11] while the incidence in other studies ranged from 0 to 20% [15–17]. In our study, 15.38% (6/39) of the patients developed preoperative endophthalmitis, and 83.33% (5/6) of them had undergone IOFB removal more than 24 h after they sustained the injury. All of them 100% (6/6) ignored the presence of the posterior segment IOFBs themselves at the moment of injury. Four among these cases did not exhibit preoperative VH but only experienced pain and blurred vision a few days following the injury, and they exhibited hypopyon and vitreous opacity during their clinic visit. Two cases, both with preoperative VH, were not suspected of having posterior segment IOFBs at first during clinical presentation until the pain continued to progress accompanied by the presence of hypopyon. We agreed that it was necessary to remove the posterior segment IOFBs as early as possible in order to prevent the development of preoperative endophthalmitis that could produce toxicity and damage the entire retina, and the same effect is produced by the IOFBs themselves, thereby leading to poor visual prognoses.

The potential limitations of our study are that we include a small number of patients, and that it was a retrospective, nonrandomized study. Nevertheless, this study on posterior segment IOFBs provides valuable and complete preoperative, intraoperative and postoperative information regarding the expected visual outcomes following IOFB-related surgical procedures.

## Conclusions

Macular injuries caused by IOFBs and postoperative RD were important related factors for poor final visual prognoses. Removing IOFB as early as possible may prevent the development of preoperative endophthalmitis, which although was not found significant to the final visual outcomes could also lead to poor final visions.

## Data Availability

This study is based in part on data from the Department of Medical Education and Research and Research Center of Medical Informatics in Kaohsiung Veterans General Hospital. The datasets used and/or analyzed during the current study are available from the corresponding author on reasonable request.

## Abbreviations

IOFB: Intraocular foreign body
VH: Vitreous hemorrhage
RD: Retinal detachment
PVR: Proliferative vitreoretinopathy
CNV: Choroidal neovascularization
SO: Sympathetic ophthalmia
CT: Computed tomography
SB: Sclera buckle
VA: Visual acuity
BCVA: Best-corrected visual acuity
PPV: Pars plana vitrectomy
